# Galactose-ƒ¿-1,3-galactose (ƒ¿-gal)-specific IgE test is highly useful for predicting cetuximab-induced anaphylaxis

**DOI:** 10.1186/2045-7022-4-S3-P52

**Published:** 2014-07-18

**Authors:** Eishin Morita, Yuko Chinuki, Hitoshi Takahashi, Makiko Takeda, Kaoru Takeuchi, Kazuyuki Ito

**Affiliations:** 1 Shimane University Faculty of Medicine, Japan; 2 Shimane University Faculty of Medicine, Department of Dermatology, Japan; 3 Matsue Red Cross Hospital, Department of Otolaryngology-Head and Neck Surgery, Japan

## Background

Cetuximab, a chimeric mouse-human IgG1 monoclonal antibody against the epidermal growth factor receptor, is approved for use in colorectal cancer and squamous-cell carcinoma of the head and neck. A high prevalence of hypersensitivity reactions to cetuximab has been reported in some areas of the United States. IgE antibodies against cetuximab were present in the patients with cetuximab hypersensitivity and the antibodies were specific for galactose-1,3-galactose (-gal) in cetuximab heavy-chain. We have also determined that patients with red meat allergy have IgE antibodies against ƒ¿-gal in Japan. In 2013, twelve patients with squamous-cell carcinoma of the head and neck were treated with cetuximab in Matsue Red Cross hospital, and found that four of twelve patients had anaphylactic shock during first infusion of cetuximab. None of them had allergic reaction to red meat before the treatment. In this study, we aimed to clarify whether the reaction was IgE-mediated or not.

## Method

Serum IgE antibodies against ƒ¿-gal were determined with CAP-fuorescent enzyme immunoassay (FEIA) (ImmunoCAP, Phadia) using bovine thyroglobulin (Sigma), and IgE antibodies against cetuximab (Bristol-Myres Squibb) were determined with immunoblotting in these patients.

## Results

Thyroglubulin-specific IgE was detected in all four patients with anaphylaxis using CAP-FEIA, whereas the eight patients who had no anaphylactic reaction had not -gal-specific IgE antibodies, indicating that sensitivity and specificity of the thyroglubulin-specific IgE test were both 100%. Anti-IgE antibodies against cetuximab were detected in sera of the four patients with anaphylaxis and two of the patients with no anaphylaxis, indicating that sensitivity of the IgE-immunoblotting was 100% and specificity of the test was 75%.

## Conclusion

CAP-FEIA test with bovine thyroglobulin (-gal) is highly useful for predicting cetuximab-induced anaphylaxis.

